# Assessing the Efficacy of Novel Fiber-Reinforced Dual-Cure Luting Resins

**DOI:** 10.4317/jced.61396

**Published:** 2024-03-01

**Authors:** Lippo Lassila, Sufyan Garoushi, Enas Mangoush, Pekka K. Vallittu, Eija Säilynoja

**Affiliations:** 1 Department of Biomaterials Science and Turku Clinical Biomaterial Center -TCBC. Institute of Dentistry, University of Turku, Finland; 2 Wellbeing Services County of South-West Finland, Turku, Finland; 3 Research Development and Production Department, Stick Tech Ltd – Member of GC Group, Turku, Finland

## Abstract

**Background:**

Dual-cure resin-based luting materials are increasingly favored in clinical applications due to their capacity to establish a strong bond with natural tooth structure and restorations. This study aimed to examine certain physical and handling characteristics of newly developed experimental dual-cure luting resins reinforced with short fibers (SFRCs) and compare them with commercially available dual-cure luting resins.

**Material and Methods:**

Seven dual-cure luting materials were tested (Relyx Ultimate, Duo-Link, eCEMENT, Variolink Esthetic, G-CEM LinkForce, experimental SFRC1, experimental SFRC2). Fourier transform infrared spectroscopy (FTIR) was utilized to determine the degree of monomer conversion (DC%) in the self and light-curing protocol. A rotating disk rheometer measured viscosity at room temperature (22°C) and simulated mouth temperature (35°C). Fracture toughness, flexural strength, and flexural modulus were evaluated using a 3-point bending test. Each luting resin was subjected to the examination of its surface microstructure using scanning electron microscopy (SEM). Analysis of variance (ANOVA) at a significance level of (*p* = 0.05) was conducted to analyze data.

**Results:**

It was revealed that DC% of the tested dual-cure resins was significantly (*p*< 0.05) affected by the curing mode, the dual-cure SFRC2 having the highest and Relyx having the lowest DC (64%, and 41% respectively). The viscosity of all tested materials decreased with increasing temperature. SFRC2 demonstrated the highest fracture toughness (2.3 MPa m1/2), while Relyx Ultimate, Duo-Link, and eCEMENT exhibited the lowest values (≈ 1 MPa m1/2)(*p*< 0.05). Both SFRCs and G-CEM link-force exhibited the highest flexural strength values, and SFRCs resulted in the highest flexural modulus values (*p*<0.05).

**Conclusions:**

The experimental fiber-reinforced dual-cure luting resins exhibited superior DC%, fracture toughness, and flexural properties, yet, SFRC2 showed the highest viscosity at elevated temperature. These results highlight the capability of short fiber reinforcement to enhance the mechanical properties of dual-cured resin-based luting materials without compromising handling characteristics.

** Key words:**Dual-cure luting resin; short fibers; degree of conversion; viscosity; fracture toughness; flexural properties.

## Introduction

In recent years, the field of adhesive dentistry has witnessed noTable advancements, leading to significant transformations in the restoration of severely compromised teeth (1). A promising shift has occurred, with clinicians increasingly emphasizing the conservation of natural tooth structure, aligning with the evolving principle of minimally invasive dentistry. Guided by biomimetic principles, the comprehensive objective now lies in the rational selection of biomaterials possessing comparable physical properties, including fracture toughness, elastic modulus, and strength, to seamlessly replace missing tooth structure (2). A widely-used biomimetic approach to dental restoration suggests using glass or hybrid ceramic to replace enamel, and short-fiber reinforced composite (SFRC) or particulate-filled conventional com-posite to replace dentine (2). This technique, supported by numerous clinical reports, typically involves creating a substructure/core build-up, in the presence or absence of an endodontic fiber post, followed by the placement of indirect restorations (3,4). In situations with substantial reduction in the structure of the endodontically treated tooth crown, using custom-made or prefabricated fiber posts, is often advised to enhance ultimate restoration retention and to improve the integrity and biomechanical properties of the residual tooth structure (4). Various types of resins are employed for luting procedures in post and indirect restoration applications. However, the toughness, elastic modulus and strength of these luting resins are typically lower than those of dentine, posts, and restorations, leading to areas of high stress. This is particularly true when a large volume or thick amount of luting resin is located in a wide or flared root canal, which can lead to the formation of multiple cracks and insufficient bonding (5).

Noteworthy, the luting resin, the weakest material used in tooth restoration, is often located in areas of the reconstructed tooth that are subjected to high tensile stress during tooth function. Furthermore, research has shown that fractures in indirect restorations typically originate from the interfacial zones between the luting resin and the ceramic surface, where the highest tensile stress is concentrated (6,7). Therefore, the use of particulate-filled luting resin does not provide optimal biomechanical properties for the restored tooth. It is unrealistic to assume that different luting resins behave differently only due to their adhesive properties, factors such as composition, mechanical, chemical, and handling properties must also be considered.

Over the past few years, a variety of luting resins with different flowability or viscosity levels have been developed by manufacturers, claiming to be suiTable for a range of applications, especially when used with fiber posts to restore teeth that have been structurally compromised due to endodontic treatment (8). Commercial luting resins are typically available in three curing modes: either self-curing, light-curing, or dual-curing. The dual-curing mode was developed to address the limited light penetrability under indirect restorations or during endodontic post placement, by combining the rapid hardening characteristics of light-curing materials with the the inherent self-curing capabilities present in self-curing materials. Most luting resins consist of methacrylate-based monomer and exhibit lower filler amount as well as mechanical performance in comparison to filling or core build-up materials (8). It is significant to note that increasing the amount of particulate-fillers can improve the mechanical characteristics of these resins, however, it would result in an increase in thickness or viscosity. The increased viscosity can compromise the integrity of the adhesive interface, leading to a reduction in bond strength and increasing the risk of microleakage (9,10). Moreover, material’s high viscosity may restrict its injectibility into the root canals, leading to the formation of gaps and voids at the interface (10,11). However, for certain post-luting materials, high viscosity may be beneficial in terms of material manipulation during core build-up procedures. Choosing the right luting resin for post-cementation is a significant challenge. The optimal approach to cementing glass-fiber posts remains matter of debate in dental practice, given the conflicting outcomes (11).

Short glass fibers reinforcement is an effective method for improving certain properties of resin composites, particularly fracture toughness (12,13). These fibers improve the material’s ability to withstand cracking, resulting in improved resilience, flexibility, and toughness (14,15). Incorporating microfibers into dual-cure luting resins presents a novel opportunity to enhance the materials’ mechanical performance. Notably, to the best of our knowledge, limited existing research has been conducted in this specific area, and no luting resin utilizing short fibers for this purpose has been previously developed or studied. Thus, the aim of this study was to assess the impact of adding short fibers on specific physical properties and handling characteristic of experimental dual-cure luting resin in comparison to commercially used luting resins. The null hypothesis evaluated was that a dual-cure luting resin may be significantly reinforced by incorporating randomly distributed microfibers.

## Material and Methods

Table 1 displays the composition of both commercial and experimental dual-cure luting resins.

**Table 1:**
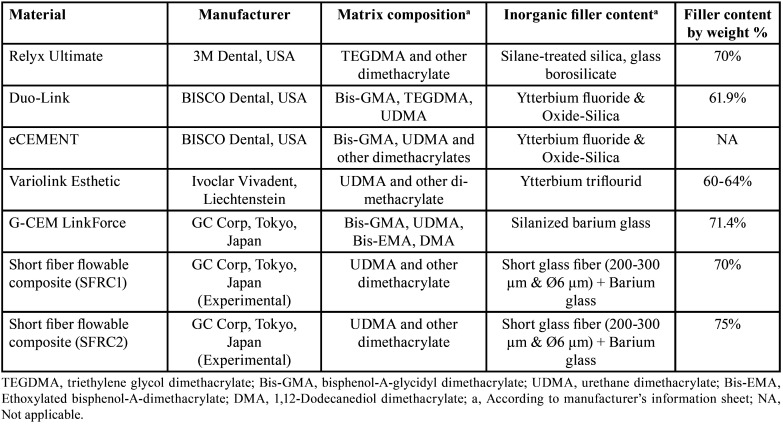
The used luting resins.

-Degree of conversion

Monomer degree of conversion (DC%) in materials underwent self-curing and light-curing protocol was determined using Fourier transform infrared spectroscopy (FTIR) equipped with an attenuated total reflectance (ATR). For analyzing the luting resins, a mold with a thickness of 2 mm and a diameter of 5 mm was used. The spectrum of the unpolymerized specimens was placed in the mold and recorded. After that, FTIR spectra for self-curing groups were acquired at one-minute intervals for 20 minutes after mixing. The self-cure mode of luting resins usually starts between 4-6 minutes from mixing, according to the manufacturer’s instructions for use (IFU). Therefore, there was a few minutes of working time. In contrast, light-curing group specimens underwent a 20-second exposure to light-curing unit visible light (D-Light® Pro, GC Europe) on a glass slide, followed by FTIR spectrum scanning. The emitted light had a wavelength of 400 to 480 nm and intensity of 1200 mW/cm2. It´s important to note that the light-curing mode employed here has a dual-cure nature, encompassing both light-curing and self-curing compo-nents. The light-curing component refers to the initiation of the polymerization process triggered by exposure to visible light. Simultaneously, the self-curing component implies a secondary mechanism wherein the material continues to cure spontaneously even in the absence of external light.

**Figure 1:**
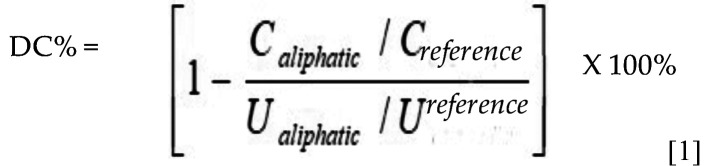
Equation 1.

The DC% was calculated by analyzing the aliphatic C=C peak at 1638 cm-1 and normalizing it against a reference peak, as per the formula below: (Fig. 1).

In equation [1], C aliphatic represents the absorption peak observed at 1638 cm–1 in the cured specimen, while ‘C reference’ denotes the reference peak of the cured specimen. Similarly, ‘U aliphatic’ corresponds to the absorption peak at 1638 cm–1 in the uncured specimen, and ‘U reference’ stands for the reference peak of the uncured specimen.

For each FTIR spectrum, the amount of double bonds that remained was determined using standard baseline techniques. This was accomplished through a comparative analysis of the peak heights corresponding to the aliphatic and reference peaks for computational purposes. Each luting resin group underwent a series of five independent trials.

-Viscosity 

To assess the viscosity of the dual-cure luting resin, rheological analysis was conducted. This involved the use of a rotational rheometer (HAAKE RheoStress 300; Thermo Electron, Karlsruhe, Germany) equipped with a parallel plate (diameter: 20 mm) and operated in dynamic oscillation mode by the application of shear stresses. Using an automix tip, the mixed dual-cure luting resin was carefully applied onto the lower plate of the rheometer, while the upper plate was meticulously adjusted to attain a uniform thickness of 0.5 mm. Any excess resin around the plate’s edge was diligently removed before measurements started.

**Figure 2:**
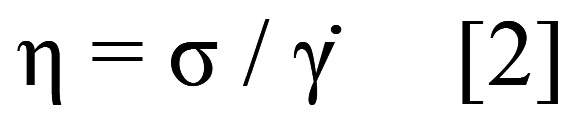
Equation 2.

The measurements were meticulously repeated three times, both at 22°C and 35°C, with each measurement extending for a duration of 300 seconds. Subsequently, the apparent viscosity of each dual-cure luting resin was calculated after a 15-second mixing period, utilizing rheological data. This calculation was grounded in the relationship defined by the equation: (Fig. 2).

In equation [2], ‘η’ denotes the apparent viscosity in pascal-seconds (Pa•s), ‘σ’ stands for the shear stress in pascals (Pa), and ‘γ̇’ represents the shear rate in seconds (s-¹), maintaining a constant value of 1.

-Fracture toughness 

Fracture toughness was measured by preparing single-edge-notched-beam specimens. The preparation of these specimens follows a modified ISO standard procedure with dimensions of (2.5 × 5 × 25 mm3). To guarantee precise dimensions and easy removal of the specimens from the mold, a custom-made split mold made of stainless steel was used, this mold facilitated effortless removal of the specimen without requiring additional force. This mold has a centrally located slot that extended to its midpoint, which allowed the notch to be positioned in the middle and optimizing the crack length to be precisely half of the specimen´s height. A single layer of the resin composite was carefully placed into the mold, which was placed on top of a glass slide that is covered with a sheet of Mylar matrix. Before polymerization, a straight-edged steel blade was intentionally inserted into the prepared slot, which resulted in the for-mation of a sharp crack. The specimens underwent polymerization, which was performend following the light-curing instructions of the D-Light® Pro device. The specimens underwent a light-curing for a duration of 20 seconds, wherein the light was applied across five overlapping rounds. To ensure uniform polymerization, the upper surface of the mold was shielded by a Mylar matrix and a glass slide on both sides of the blade. Subsequently, the specimens were stored under controlled dry conditions at 37°C for a period of 48 hours prior to testing.

The calculations of fracture toughness were carried out using a well-established specific formula [3]. All the specimens were subjected to evaluation through a three-point bending mode test, using a universal testing machine. The testing procedure was applied at a controlled crosshead speed of 1.0 mm/min.

**Figure 3:**
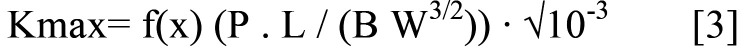
Equation 3.

The used formula for calculating fracture toughness [3], denoted as ‘Kmax,’ is expressed as a function of ‘x,’ as follows: (Fig. 3).

wherein ‘f(x)’ is defined as 3/2x^(1/2) (1.99 - x(1 - x)(2.15 - 3.93x + 2.7x^2)) / 2(1 + 2x)(1 - x)^(3/2), with ‘x’ being a geometric function dependent on the ratio of crack length (‘a’) to specimen width (‘W’), and constrained within the range 0 < x < 1, where ‘x’ is defined as ‘a/W.’

In the context of the provided equation [3], ‘P’ represents the maximum load, expressed in Newtons (N), ‘L’ signifies the span length, measured at 20 mm, ‘B’ represents the specimen´s thickness in mm, ‘W’ represents the width (or depth) of the specimen in mm.

-Flexural strength and modulus of elasticity

**Figure 4:**
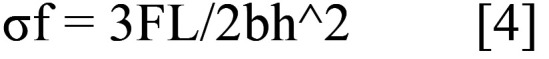
Equation 4,5.

**Figure 5:**
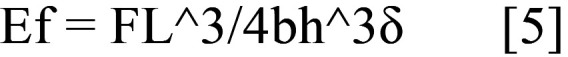
Equation 4,5.

Bar-shaped specimens were fabricated using a stainless-steel mold and transparent Mylar matrix sheets, with dimensions of 2 x 2 x 25 mm³. The luting resin was polymerized using a hand light-curing unit (D-Light® Pro) for a duration of 20 seconds. The curing process involved applying the light in five overlapping sections on both sides of the mold, with a light tip distance of 1 mm. The specimens were stored dry at 37°C for 48 hours before testing. A three-point bending test was conducted according to the ISO 4049 standard, with a test span of 20 mm, crosshead speed of 1 mm/min, and indenter diameter of 2 mm. The load-deflection curves were recorded using a material testing machine (model LRX, Lloyd Instrument Ltd, Fareham, England) and a computer software (Nexygen 4.0, Lloyd Instruments Ltd, Fareham, England). 8 specimens were tested for each luting resin group. The flexural strength (σf) and flexural modulus (Ef) were determined using the following mathematical formulas (16): (Figs. 4,5).

In equations [4,5] the variables are defined as follows: ‘F’ represents the load at fracture in newtons (N), ‘L’ signifies the span length, ‘b’ denotes the specimen width, ‘h’ represents the specimen thickness, and ‘δ’ indicates the deflection at fracture, all measurement were in millimeters.

-Microstructure analysis

Scanning electron microscopy (SEM) was used to examine the microstructure of the dual-cure luting resins and EDS (GeminiSEM 450, Carl Zeiss, Ober-kochen, Germany) was used for characterization purposes. After being polished, the composite specimens were kept for 24 hours in a desiccator under dry conditions. Subsequently, specimens were gold coated with a sputter coater installed in a vacuum evaporator (BAL-TEC SCD 050 Sputter Coater, Balzers, Liechtenstein). Gold coating was applied to facilitate SEM/EDS examination. SEM observations were conducted using 20 kV operating voltage and 10 mm working distance.

-Statistical analysis

The data obtained from the study were analyzed statistically using the two-way ANOVA test. This was followed by the one-way ANOVA and Tukey HSD test with a significance level of 0.05, in order to determine the differences between the groups. The statistical analysis was conducted using the SPSS version 23 software, developed by IBM Corporation, New York, USA.

## Results

**Figure 6:**
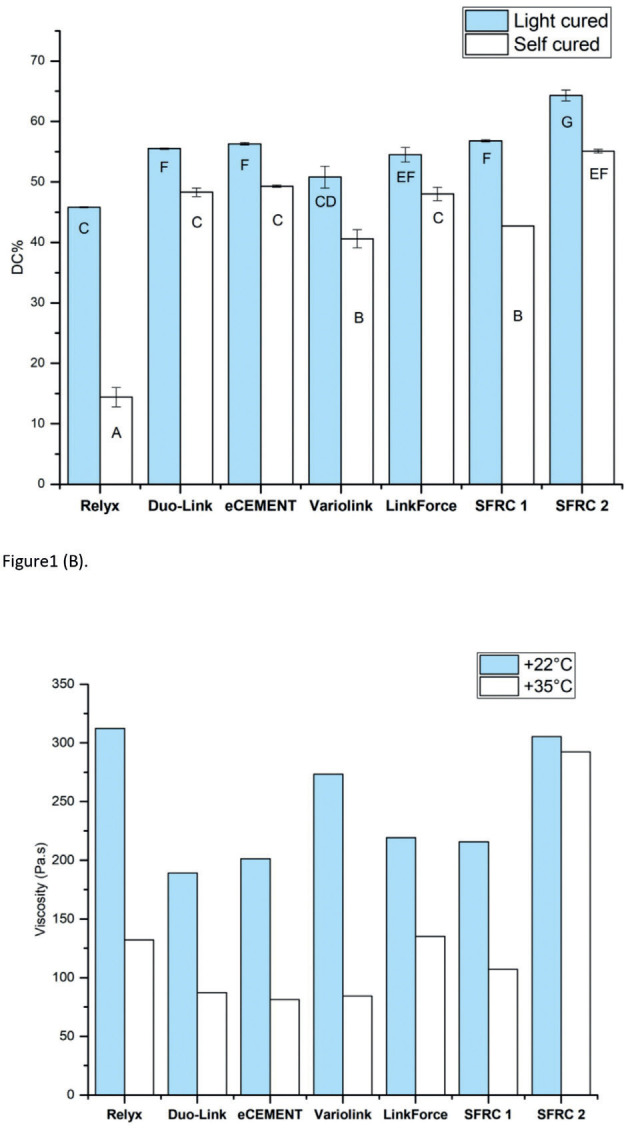
(A) Graph displays the average values for degree of monomer conversion (DC%) of tested dual-cure luting resins, along with their corresponding standard deviations (SD). Groups with different letters have statistical significant differences from each other (significance level *p*<0.05). (B) Graph dis-plays the average values for viscosity (Pa.s) of evaluated dual-cure luting resins utilizing rotational rheometer at 15 seconds following mixing under different temperatures.

The results showed that the DC% of the luting resins increased significantly (*p*<0.05) when a light-curing protocol was used, as seen in Figure 6A. The DC% of the evaluated materials ranged between 40 and 64%, except for Relyx Ultimate, which had a very low value of 14.5 after the self-curing protocol (Fig. 6A).

The viscosity of all tested materials decreased with increasing temperature (Fig. 6B). Viscosity in SFRC2 increased noticeably compared to the other materials at both measuring temperatures.

**Figure 7:**
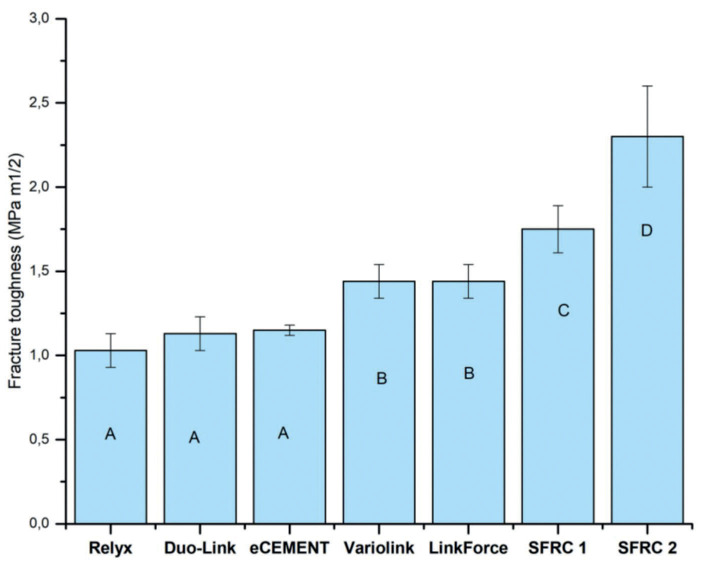
Graph displays the average values for fracture toughness (KIC) of evaluated dual-cure resin composites, along with their corresponding standard deviations (SD). Groups with different letters have statistical significant differences from each other (significance level *p*<0.05).

The fracture toughness of the tested materials is shown in Figure 7. Experimental SFRC2 had the highest fracture toughness values (*p*<0.05) at 2.3 MPa m1/2. Relyx Ultimate, Duo-Link, and eCEMENT had the lowest values, approximately 1 MPa m1/2.

**Figure 8:**
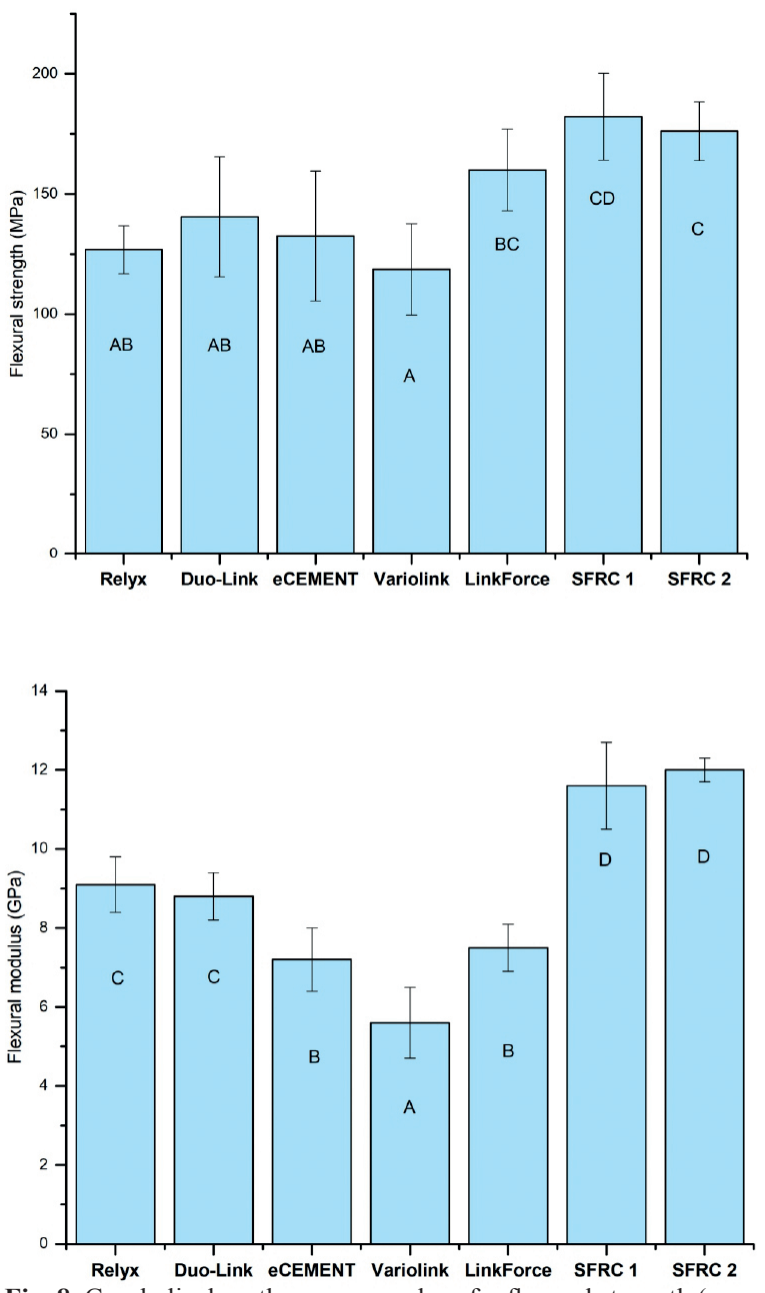
Graph displays the average values for flexural strength (measured in MPa) and flexural modulus (measured in GPa) of tested dual-cure resin composites, along with their corresponding standard deviations (SD). Groups with different letters have statistical significant differences from each other (significance level of *p*<0.05).

The outcomes of the flexural properties (flexural strength and modulus) of the tested dual-cure luting resins are presented in Figure 8. Statistical analysis using ANOVA indicated that the type of material significantly affected the tested flexural properties (*p*<0.05). SFRCs and G-CEM linkForce materials exhibited the highest flexural strength values, while the other luting resins had comparable values. Variolink exhibited the lowest flexural modulus values, and SFRCs resulted in the highest values (*p*<0.05). There was no statistically significant difference between the flexural properties of SFRC1 and SFRC2 (Fig. 8).

Table 2 displays the findings of the investigation of the major elements existent in each tested luting resin, which were determined using EDS.

**Table 2:**
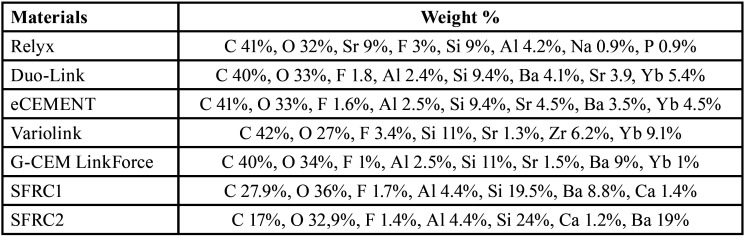
Elements analysis of tested resin composites.

**Figure 9:**
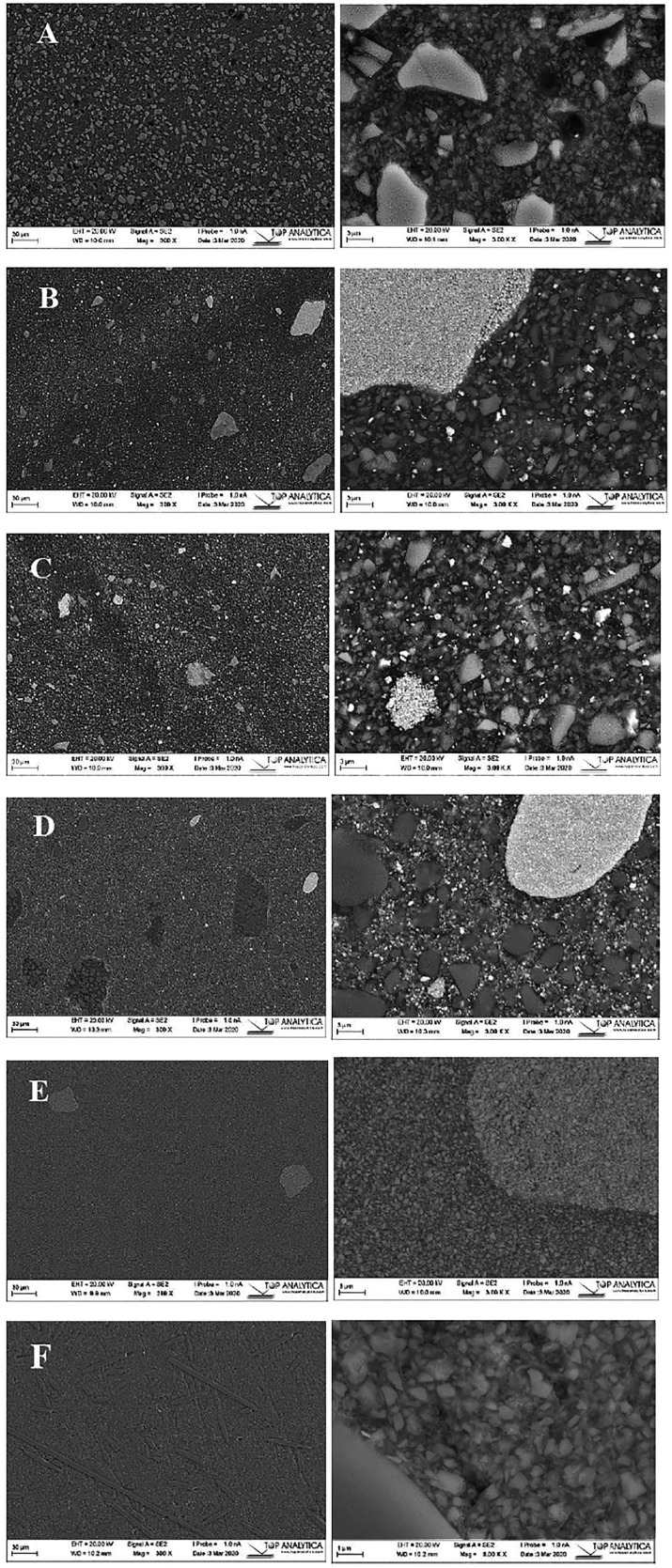
SEM images (magnification: 300 & 3000 X) of polished surface of investigated commercial luting resins (scale bar = 30, 3µm). (A) Relyx; (B) Duo-Link; (C) eCEMENT; (D) Variolink; (E) G-CEM LinkForce, and investigated experimental luting resin reinforced with short fibers (F), (magnification: 300, 9000 X).

Figure 9A-F showed that the SEM analysis demonstrated smooth and uniform surfaces where fillers were evenly distributed within the resin matrix of commercial and experimental SFRC luting resins. The specimens exhibited variations in terms of the size, shape, and quantity of particulate fillers, which could explain the differences in the toughening and reinforcing capacity observed among the tested materials.

## Discussion

The present study investigated the properties of dual-cure luting resins, emphasizing the impact of short fiber reinfircment, curing protocols, filler content, and temperature on various physical and handling characteristics. The results of this study support the hypothesis that microfibers can still have a significant effect on flexural strength and fracture toughness together with the easy to use properties of a luting material.

The findings of the present study indicate that the curing protocol significantly influenced the degree of conversion (DC%) in the tested luting resins, as shown in Fig. 9A. Our results align with prior research findings (17-19), which indicated that the risk of displacement and prolonged setting time must also be considered when relying solely on a self-curing protocol. Despite observing a decrease in DC% for most materials under the self-curing mode, they remain viable choices for challenging areas like the root canal. It’s worth noting to consider that our measurements were only taken for 20 minutes after mixing, and literature suggests that DC% increases over time through continuous polymerization reactions (20).

Understanding how temperature and the specific type of luting resin can influence the precision of DC% measurements is crucial. Several variables, such as the type and concentration of initiators and co-initiators, the presence of inhibitors, and the type and quantity of fillers, can impact the rate and degree of polymerization, ultimately affecting the DC% of luting resins (21). Differences in specimen preparation, testing equipment, and measuring conditions can also influence the accuracy and reproducibility of DC% measurements. In Table 1, some dual-cure luting resins were UDMA-based, while others were Bis-GMA-based. To measure the DC% of UDMA-based resins, a different reference peak needs to be selected due to the absence of an aromatic ring in their structure. In the current study, the DC% of UDMA-based luting resins was evaluated by measuring the secondary amide peak at 1530 cm-1 (21,22). Therefore, carefull interpretation and comparison of DC% results from different studies is necessary, considering the limitations and potential sources of error associated with the experimental methods used. The results showed that Relyx Ultimate exhibited the lowest DC% and frature toughness compared to the other tested materials. This phenomenon can be explained by the distinctive chemical composition of Relyx Ultimate, it is the only examined luting resin lacks UDMA monomer. It composed mainly of TEGDMA (Table 1) which is known for its lower viscosity and higher flexibility, making it less reactive than UDMA (16,23). UDMA is recognized as a flexible monomer plays an important role in facilitating the cross-linking mechanism during the polymerization process. Consequently, its absence in Relyx Ultimate contributes to a diminished degree of conversion and, consequently, a reduction in the material’s fracture toughness (16). Furthermore, the lower DC% observed in RelyX Ultimate, particularly during self-curing, can be atributed to the presence of acidic-functional monomers in its composition. This inclusion may impede polymerization through a peroxide-amine binary redox system when employing the self-curing protocol (17). Additionally, the lower DC% in the dual-cure mode could be explained by the competition between the acid-base reaction and the resinous polymerization reaction during the curing process, which is capable of retarding the DC% (16). The experimental SFRCs luting resins have comparatively high DC% values, this can be credited to the presence of incorporated glass fibers, which possess the capability to efficiently transmit light. Consequently, this efficient light transmission contributes to an enhanced rate of monomer conversion values (24)

The viscosity of dual-cure luting resins was assessed using a rotational rheometer test at room temperature and at 35°C, simulating oral cavity conditions. The filler content could also influence the viscosity, with SFRC2 having a higher filler/fiber content compared to other tested materials, resulting in a higher viscosity (Fig. 6B). To the contrary, Duo-Link showed the lowest viscosity values since it has lower filler content (Table 1). SFRC1 contains fillers constituting 70% of its weight, while SFRC2 contains fillers constituting 75% of its weight. The increased viscosity of SFRC2 may offer advantages during core-build up but could also pose challenges in material manipulation.

The temperature can also affect the viscosity of the materials. When the materials were in a fluid paste state immediately after mixing, the viscosity decreased due to formation of flexible molecular networks (25). This phenomenon was observed in SFRC1, where the viscosity was halved with an increase in temperature to 35°C. However, shortly after, it became apparent that the impact of temperature on the catalytic acceleration of the polymerization reaction outweighed its influence in decreasing viscosity.

Fractures in resin-based composites commonly occur in various clinical applications (26,27). Micro-cracks can initiate and propagate through the material under occlusal stresses, resulting in material or luting failure. Fracture toughness, a mechanical property, evaluates the capacity of brittle materials to resist crack propagation under applied stress, it characterizes the material’s ability to withstand damage and serves as a measure of its resilience against fatigue (2). Previous studies indicate that conventional resin-based materials are brittle, rendering them susceptible to bulk fracture that can propagate easily through the entire material and even reach the tooth structure (28). By introducing reinforcing elements like fibers, there is promising evidence of improving the mechanical properties of the material. This suggests a potential pathway for enhancing the toughness and durability of restorative and luting materials (29). Incorporating fibers, that work as energy absorber and stress distributer, can redirect the propagation of cracks away from the main body of the material and towards its edges (30,31).

Our results confirm that the dual-cure SFRCs exhibited the utmost fracture toughness values compared to the other luting resins tested (Fig. 7). Interestingly, SFRC2, which has higher fiber/filler loading, showed a significantly higher value (2.3 MPa m1/2) of fracture toughness in comparison with SFRC1 (1.7 MPa m1/2). The capability of fiber reinforcement is influenced by several parameters, involving the type of resin used, fiber weight, orientation, placement, aspect ratio, fiber-matrix adhesion, and fiber impregnation into the resin matrix (17). On the other hand, Variolink Esthetic and G-CEM LinkForce exhipited higher fracture toughness than other commercial luting resins, owing to their matrix flexibility.

The three-point bending test is a commonly used methodology in dental composite research due to its inherent advantages in uncomplicated specimen preparation and uniform load application. Among the tested dual-cure luting resins, G-CEM LinkForce and SFRCs exhibited the highest flexural strength values, which could be attributed to their high filler/fiber content in comparison to the other tested luting resins. G-CEM LinkForce is known for its high barium glass filler rate (71.4 wt%), while SFRCs have a filler/fiber reinforcement rate of 70-75 wt% (21,32). The SFRCs were anticipated to have greater flexural modulus values because of their appropriate fiber aspect ratio and ability to provide reinforcement (32). Conversely, Variolink Esthetic, with a comparatively less filler content (60-64 wt%), exhibited the lowest flexural modulus, measuring less than 6 GPa. This emphasizes a consistent correlation observed between flexural properties and the inorganic filler loading of the investigated luting resins aligning with findings from prior studies (9,16,33). Nevertheless, factors other than filler loading may contribute to the variations in flexural properties among the materials. These factors may include variations in filler characteristics such as type and size, composition of the resin matrix, quality of filler silanization, or the presence of structures that resist cracking, such as nano-clusters of fillers or fibres (34).

The EDS analysis findings showed only minor differences in the oxides of the tested luting resins (Table 2), assuming that the observed variability in performance could be predominantly attributed to the complicated interaction of the morphological features and spatial distribution patterns of the reinforcing fillers within the resin matrices. As mainly the larger particles were examined, no definitive predictions can be drawn solely depended on these results. SEM images (Fig. 9A-E) of the tested commercial materials reveal a uniform distribution of fillers with various sizes, shapes, and quantities within the resin matrix. In Fig. 9F, a homogeneous mixture of fillers and fibers of different lengths is detected. The images display a well-integrated structure where the fibers closely adhere to the surrounding matrix, and there are no detecTable gaps.

Interestingly, recent study by Suni *et al*. reported that the use of commercial flowable light-cured SFRC (everX Flow) as luting resin with endodontic fiber posts proved to be a encouraging technique to improve the interface adhesion (35). According to their findings, SFRC has higher bond strength values (23.5 MPa) than conventional dual-cure luting resin (G-CEM LinkForce) values (12.5 MPa). Their findings are explained by the micro-mechanical interlocking mechanism that occurs between the exposed short fibers on the interface surfaces with the fiber post. Additionally, the fracture toughness values of the luting SFRC resin would play a crucial role in enhancing the resistance capacity against shearing stresses.

To achieve a more comprehensive understanding of the properties of the new experimental dual-cure SFRCs and to address specific research gaps, additional investigations are required. These should include an examination of polymerization shrinkage characteristics, such as volumetric shrinkage and shrinkage stress. Additionally, parameters such as film thickness, adaptation, microleakage, and bonding strength to dentin, fiber post, and indirect restoration warrant further exploration.

## Conclusions

1. Light-curing protocol significantly increased the degree of conversion (DC%) of luting resins.

Among the evaluated materials, dual-cure SFRC2 has the highest conversion rate (64%).

2. The viscosity of all tested materials decreased with increasing temperature, dual-cure SFRC2, with a higher filler/fiber content, showed a noticeable increase in viscosity compared to other materials at both measuring temperatures.

3. Experimental dual-cure SFRC2 displayed the highest fracture toughness value (*p*<0.05) at 2.3 MPa m1/2, while Relyx Ultimate had the lowest value, approximately 1 MPa m1/2.

4. Type of material significantly influenced the tested flexural properties (*p*<0.05). Dual-cure SFRCs and G-CEM link-Force materials exhibited the highest flexural strength values.

5. SEM analysis demonstrated smooth and uniform surfaces with even distribution of fillers within the resin matrix of the commercial and experimental SFRC luting resins. Specimens exhibited variations in the size, shape, and quantity of particulate fillers, which could explain observed differences in toughening and reinforcing capacity among tested materials.
